# Coherent Noise Suppression Using Adaptive Homomorphic Filtering for Wideband Electromagnetic Imaging System

**DOI:** 10.3390/s19204469

**Published:** 2019-10-15

**Authors:** Yanju Zhu, Shuguo Xie

**Affiliations:** School of Electronic and Information Engineering, Beihang University, Beijing 110191, China; zhuyanju1309@163.com

**Keywords:** coherent noise, adaptive homomorphic filtering, MDL, wideband electromagnetic imaging system

## Abstract

The wideband electromagnetic imaging system using a parabolic reflector is a device for detecting and locating electromagnetic interference sources (EMIS). When multiple coherent interference sources are detected, the confusion will occur due to the coherent noise that is caused by interference phenomenons. Previous works have removed the coherent noise by using iterative techniques, but they face a limitation in removing noise in that the coherent noise pattern changes with frequency in a wideband. In this paper, an adaptive homomorphic filtering is proposed to overcome the limitations of conventional methods from 1 GHz–6 GHz. The coherent noise existing in the several electromagnetic images is studied, and it is confirmed that the variation of the coherent noise pattern is related to the position, the number, and the frequency of EMIS. Then, by analyzing the probability density of coherent noise intensity, an adaptive Gaussian filter is carefully designed to remove coherent noise. The filter parameters are selected by the minimum description length criterion (MDL) to apply to compute directly the local amount of Gaussian smoothing at each pixel of each image. The results of the experiments and simulations demonstrate that the proposed method can significantly improve the quality of electromagnetic images in terms of maximum sidelobe level (MSL) by 15 dB and dynamic range (DR) of the system over 20 dB, compared with conventional narrowband denoising methods.

## 1. Introduction

With the development of edge computing and interest of things technologies, electronic devices are being widely used in various applications. Since some of these devices are operating at the same frequency points and emitting wideband electromagnetic signals, these node devices may also become same-frequency interference sources. A spectrum analyzer or other qualified measurement equipment is used to detect electromagnetic interference sources (EMIS). However, it is very time-consuming to use these methods, which require a series of complex and direct measurements on the device surface at different times. A wide-range, wide-band, and far-field [1] electromagnetic imaging system is proposed to measure field distribution to the plane of the device and locate EMIS in real-time. When the multiple coherent interference sources are detected, the confusion could occur due to coherent noise that is caused by interference phenomenons and the interference intensity of the diffraction wave may be stronger than the intensity of the EMIS. Therefore, it leads to low spatial resolution and false source identification in localization of the multiple sources. Moreover, the interference intensity of the diffraction wave changes with the position, the number, and the frequency of EMIS, leading to variation in the coherent noise pattern. Therefore, the removal of coherent noise from electromagnetic images is a very challenging task.

Coherent noise arises in many practical applications such as laser, microscope, ultrasound images, and synthetic aperture radar (SAR) images, which use coherent demodulation of reflected electromagnetic waves. The additive noise model is not suitable to express the coherent property of these image acquisition processes. The multiplicative noise model is more appropriately applied to these coherent imaging systems, compared with the additive noise. The popular multiplicative noise removal methods include transforming domain-based, non-local filtering and variational methods. The transform domain-based method has attracted much attention, since it can extend multiplicative noise removal methods to the direct application of most state-of-the-art algorithms. José M. Bioucas-Dias [2] used the logarithmic transformation to convert the multiplicative model into an additive one and applied variable splitting to obtain an equivalent constrained problem. JianLu [3] proposed a variational model formulated in the logarithm transforms domain of the desirable images for the restoration of images corrupted by multiplicative noise. A new method was proposed by Jing Dong [4] using a sparse analysis model that contains a data fidelity term and two regularizes to remove the multiplicative noise. However, these methods are iterative and thus do not allow one to predict the convergence process at all and are not applicable to real-time measurements. Norashikin [5] used a subspace-based spatial domain constraint method (SDC), which applies a homomorphic framework in order to convert multiplicative speckle noise into an additive, for speckle noise removal from SAR images. Devanand [6] used the logarithmic transform and bivariate thresholding-based dual-tree complex wavelet transform to remove additive and multiplicative noise. However, most of the wavelet thresholding methods suffer from the drawback that the predefined thresholds may not match the specific distribution of signal and noise components in different scales. Recently, some new approaches have been proposed. Charles [7] proposed a general scheme to include the Gaussian denoisers within a multi-channel SAR speckle reduction technique called MuLoG (MLG). Weiying Zhao [8] proposed a fast and efficient multitemporal despeckling method by using the ratio image provided by the ratio between an image and the temporal mean of the stack. The wide-band and space-variant property of the imaging system ensures the variation of the coherent noise pattern. Existed methods cannot cope with the problem stated above.

In this paper, we analyze that the coherent noise pattern changes with the position, the number, and the frequency of EMIS, and find out the filter parameters that are valid for coherent noise removal. To meet real-time, accurate, and adaptable requirements of the imaging system, an adaptive Gaussian filter is designed to enable coherent noise to be eliminated after taking logarithms. The filter parameters are selected by MDL to directly compute the local amount of Gaussian smoothing in each pixel of each image. Our technique is not iterative, it is very stable and does not require any thresholds. Therefore, it can realize real-time processing on terminal equipment.

## 2. Preliminary Foundations 

To estimate the coherent noise accurately, the key issue is to find out the properties of the coherent noise components and distinguish them from the signal using an appropriate method. The variation of the coherent noise pattern regarding the position, the number, and the frequency of EMIS is discussed, and then the filter parameters that are valid for coherent noise removal are discussed.

### 2.1. Feature Analysis of Coherent Noise

Following the Fraunhofer-diffraction theory, the expression for the composite image intensity distributions from two-point sources formed by the circular pupil function in the image plane of the coherent optical systems are given by [9], as follows:

(1) $$I_{o}(Z)={\left|P(Z-Q)\right|}^{2}+\alpha {\left|P(Z+Q)\right|}^{2}+2\sqrt{\alpha \mu (Z_{0})}\left|P(Z-Q)\right|\left|P(Z+Q)\right|$$

The two-point sources are separated by the distance 2*Q* = *Z*_0_; α is the intensity difference between the two coherent sources; *μ*(*Z*_0_) is the substantial part of the complex degree of the coherence of the illumination; *Z* is the dimensionless diffraction variable; and *P*(*Z* + *Q*) and *P*(*Z* − *Q*) are the amplitude impulse responses of the coherent optical imaging system corresponding to the two-points, positioned at a distance of *Z*0/2 on either side of the optical axis. In the electromagnetic imaging system, *I_s_* is the image intensity distribution of the two EMIS, *a* = 1 gives the case of equal intensities of the two sources, and *I_s_*_1_ and *I_s_*_2_ are the intensity impulse responses of the imaging system corresponding to the two sources, respectively. According to Equation (1), the coherent noise can be expressed as follows:

(2) $$I(x,y)=I_{s}(x,y)-I_{s1}(x,y)-I_{s2}(x,y)$$

Some of the properties of coherent noise in the electromagnetic images have been studied. The coherent noise is integrated with a finite aperture; the probability distribution of the noise intensity has been given by Goodman [10], and is expressed as

(3) $$p_{I}(I)=\frac{M^{M}}{\Gamma (M)I_{0}}{\left(\frac{I}{I_{0}}\right)}^{M-1}\exp(-\frac{MI}{I_{0}}),$$

where *I*_0_ is the mean intensity and *M* may be interpreted as the effective number of the noise in the integrating aperture. The parameter *M* is closely related to the space-bandwidth product *R* of the imaging system–detector combination. For example, in the case of a square pupil function of side *Dp* and a square scanning aperture of side a,

(4) $$R={\left(aD_{p}/\lambda d\right)}^{2},$$

where *d* is the distance between the exiting pupil and the image plane and λ denotes the wavelength of the coherent source used. According to [11], it may be shown that the expression for *M* is then

(5) $$M={\left(2\int_{0}^{1}\left(1-\tau )\sin c^{2}(\sqrt{R}\tau \right)d\tau \right)}^{-2},$$

so that

(6) $$M≃\begin{array}{c} R\begin{array}{cc} & for\begin{array}{cc} & R\gg 1, \end{array} \end{array}\\ 1\begin{array}{cc} & for\begin{array}{cc} & R\ll 1. \end{array} \end{array} \end{array}$$

Consider a noise image to which the transformation *D* = − ln(*I*) is applied. The probability distribution (3) is then transformed into

(7) $$f_{D}\left(D\right)=[M^{M}/\Gamma (M)]\exp[-M(D-D_{0})]\exp\left\{-M\exp[-(D-D_{0})]\right\}$$

where *D*_0_= −ln(*I*_0_). According to [12], if M ≫1, and the number *M* of noise in the aperture is large enough, the log-transformed noise is approximately Gaussian additive noise with a variance equal to 1/*M*. For small values of M, this is not true. For values as small as *M* = 3, the Gaussian approximation for *D* is seen to be relatively good.

In the electromagnetic imaging system, the interference source (1 GHz–6 GHz) imaging is 10 m away from the reflective surface. An offset paraboloid with a diameter of 3 m can realize a wide range of imaging, corresponding to 5.2 m × 2.6 m in rectangular area. The far-field maps are visualized from the scan plane delimited by 1.2 m × 0.75 m. The distance d between the exit pupil and the image plane is 2.05 m. In the imaging system, *a* = 1.2 m, *D_p_* = 3 m, *d* = 2.05 m, the wavelength of the electromagnetic wave is from 0.05m to 0.3m, then the values of R and M are from 34.265 to 1233.55. Therefore, the higher the frequency of the electromagnetic interference source, the closer the distribution of the logarithm of the coherent noise to the Gaussian distribution.

**Figure 1:**
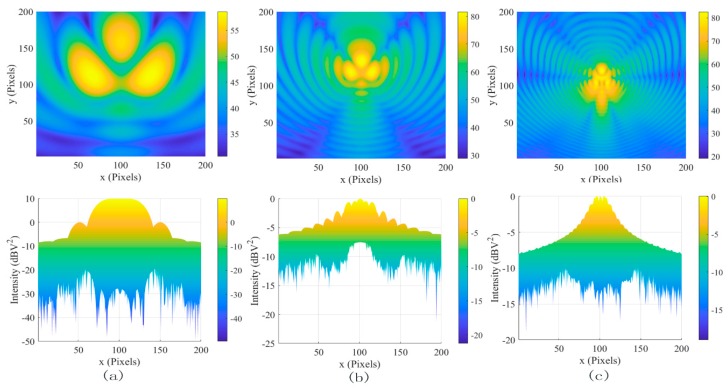
The images of coherent interference sources and the distribution of the logarithm of the coherent noise intensity in the images. (**a**) At 2 GHz, (**b**) At 4 GHz, and (**c**) at 6 GHz.

**Figure 2:**
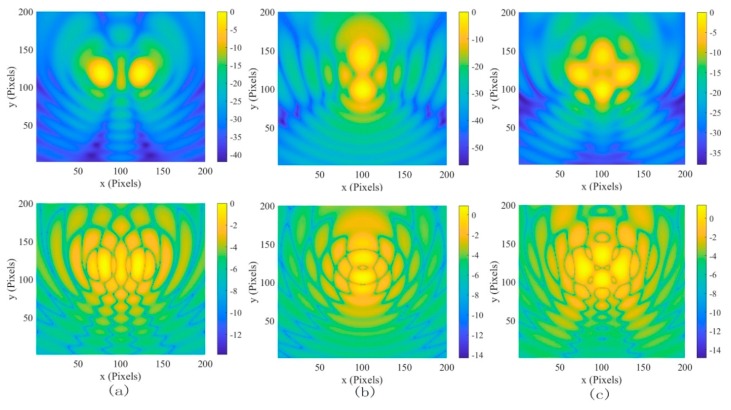
The images of coherent interference sources and the distribution of the logarithm of the coherent noise intensity in the images at 2 GHz. (**a**) Two coherent electromagnetic interference sources (EMIS) placed horizontally, (**b**) two coherent EMIS placed vertically, and (**c**) four coherent EMIS.

The interference sources at 2 GHz, 4 GHz, and 6 GHz, and the distribution of coherent noise intensity in the images, are shown in Figure 1. The probability density of the logarithm of the coherent noise intensity gets closer to the Gaussian density as the frequency increases, and the dynamic range of the system is from −40 dB to −18 dB. Moreover, the distribution of coherent noise is not only related to frequency, but also it is affected by the number and position of the interference sources. The electric field distributions of different numbers of EMIS at different positions are shown in Figure 2; the probability density of the logarithm of the coherent noise intensity in the images has changed, and the dynamic range of the system is from −15 dB to −13 dB. Owing to this large variation in electromagnetic wave path lengths among many possible electromagnetic wave paths along which electromagnetic waves travels, the frequency sensitivity of the coherent noise can be significantly enhanced.

### 2.2. Selection of Parameters

**Figure 3:**
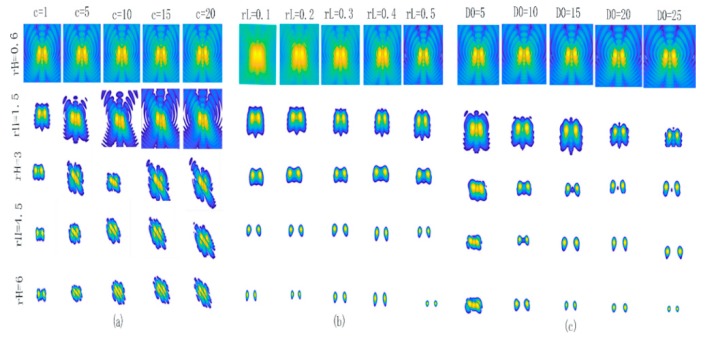
The image representation of the homomorphic filter for five different $r_{H}$ values for two dipole sources at 3 GHz. (**a**) Against five different c values, (**b**) against five different $r_{L}^{}$ values, and (**c**) against five different *D*_0_ values.

The homomorphic filtering process is composed by a natural logarithmic transformation, and the frequency filter has circularly symmetric curve shape, with its center having (*u*,*v*) = (0,0) coordinates. The transfer function of the filter, which is modified by Gaussian high-pass filter, is expressed as follows:

(8) $$\begin{array}{ll} H\left(u,v\right) & =\left(r_{H}-r_{L}\right)\left[1-\exp\left\{-c{\left(\frac{D\left(u,v\right)}{D_{0}}\right)}^{2}\right\}\right]+r_{L}\\ & =(r_{H}-r_{L})[1-\exp(-c\frac{D^{2}(u,v)}{D_{0}^{2}})]+r_{L}\\ & =r_{H}[1-\exp(-c\frac{D^{2}(u,v)}{D_{0}^{2}})]+[\exp(-c\frac{D^{2}(u,v)}{D_{0}^{2}})]r_{L} \end{array}$$

where constant *c* is applied to control the steepness of the slope, *D*(*u*,*v*) is the distance between coordinates (*u*,*v*) and the center of frequency at (0,0), and *D*_0_ is the cut-off frequency. The high frequency gain $r_{H}$ and the low frequency gain $r_{L}^{}$ are two adjustable parameters, expressed as follows:

(9) $$r_{H}>r_{L}\geq 0$$

where $r_{L}^{}$ is the low frequency gain which has a small variable range was from 0 to 1. Based on (8), a smaller $r_{H}$ will realize more severe inhibition of the low frequencies, and a larger $r_{H}$ will lead to greater enhancement of the high frequencies. The *D*_0_ is the cut-off frequency in the Gaussian filter that controls the amount of information in the scale-space. These make $r_{H}$ and *D*_0_ the most important parameters of the system. EMIS are detected and located from 1 GHz to 6 GHz by the electromagnetic imaging system, and the image of two interference sources at 3 GHz is selected to explore the filter parameters to influence on coherent noise. Figure 3 shows the image representation of the homomorphic filter for different $r_{H}$, *D*_0_, $r_{L}^{}$, and *c* values. It clearly shows that coherent noise reduces gradually with changing values of $r_{H}$ and *D*_0_, and that the spatial resolution improves. However, the coherent noise removal does not change much with changing values of $r_{L}^{}$ and *c*. This indicates that $r_{H}$ and *D*_0_ are the important parameters in our system. On the other hand, the position, the number, and the frequency of the EMIS are unknown and require a different filter to reduce coherent noise in the image. Therefore, we produce the adaptive homomorphic filter based on each input image by adjusting the parameters $r_{H}$ and *D*_0_. In our experiment, to avoid the intensity information of electric field being damaged, the filter parameters are empirically set to be *c* = 1 and $r_{L}=0.5$.

## 3. Proposed Method

**Figure 4:**
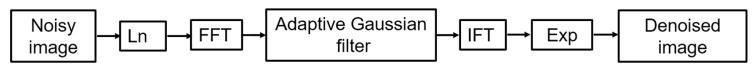
The proposed adaptive homomorphic system.

The structure of the proposed adaptive homomorphic system to reduce coherent noise is shown in Figure 4. First, the natural logarithmic function is used to transform the input coherent noisy image to an additive one, and then FFT is applied. In the frequency domain, consider a stack of smoothed images; the appropriate filter parameters are chosen by a set of Gaussian kernels. The optimal coding is the selection criterion, and it can be estimated with tools using the MDL. After that, an image is made the inverse Fourier transform (IFT). Exponential function is then applied to the output to get an estimate of the denoised image. The adaptive Gaussian filter parts of the proposed system are explained by the next few sections.

### 3.1. Adaptive Gaussian Filter

In frequency domain, a stack of smoothed images can be expressed as $I_{p1},I_{p2},\ldots I_{p3}$ which are obtained by multiplying the initial image *I*_0_ with a set of Gaussian kernels $G_{p},p=p_{1,\ldots ,}p_{n}$, where $I_{p}=I_{0}\times G_{p}$. Thus, it is important to choose the appropriate $r_{H}$ and *D*_0_; the optimal coding is the selection criterion that is effective for describing the parameter. In practice, tools like MDL [13] are used to estimate the optimal code. An image after applying a Gaussian filtering is

(10) I0(u,v)=Ip(u,v)⏟High−Pass+ε(u,v)⏟Residual

where the original image minus the smoothed image is the residual *ε*. In machine vision for balancing simplicity and accuracy, the idea of selecting the MDL has been successfully applied. In an electromagnetic imaging system, the coherent noise is eliminated by adjusting the filter parameters. Optimal coding can obtain the minimum number of bits and the maximum information of the filter parameters, that is, the maximum smoothness with the minimal residual. Equation (10) is rewritten as description length (*dl*), as follows:

(11) $$dl_{I_{0}(u,v)}=dl_{I_{p}(u,v)}+dl_{\varepsilon_{}(u,v)}$$

### 3.2. Description Length of $I_{p}$ and $\varepsilon_{}$

The $r_{H}$ and *D*_0_ are used to compute a fast approximation of the amount of information in $I_{p}$. The cut-off frequency *D*_0_ is the half power point where the filter response is reduced to 0.5 (−3 dB) in the power spectrum or $1/\sqrt{2}\approx 0.707$ in the amplitude spectrum and $r_{H}$ is used to control the amplitude spectrum of the filter. 

Furthermore, $r_{H}\propto D_{0}$, when a constant (β) is given, $r_{H}$ can be expressed as $r_{H}=\beta D_{0}$. The sampling theorem states that for any signal of frequency f, the number of samples s (Nyquist rate) needed for reconstructing accurately the original signal is 2f at least. As we know, $f\propto D_{0}{}^{2}$; the frequency *f* is proportional to the Gaussian filter band-width, which is controlled by $r_{H}{}^{2}$. Given a constant (α), the sampling rate could be expressed as $s=n(\alpha \beta^{2}r_{H}{}^{2}),n\geq 2.$

Although the bits representing each *s* are unknown, they are proportional (*s* ∝ bits) to the amount of information, given a constant μ. We can express $I_{r_{H}}$ as follows:

(12) $$dl_{I_{r_{H}}(u,v)}=n(\mu \alpha \beta^{2}r_{H}{}^{2});n\geq 2$$

According to [14], the *D*_0_ in the Gaussian filter controls the amount of information in the scale-space; the description length of the parameter *D*_0_ of the Gaussian filter is estimated in bits as follows:

(13) $$dl_{I_{D_{0}}(u,v)}=n(\mu \alpha D_{0}{}^{2});n\geq 2$$

The DML in bits of a smoothed image given the $r_{H}$ and *D*_0_ of the Gaussian filter are estimated by Equations (12) and (13). In addition, Equations (12) and (13) demonstrate that a correct description length of $I_{r_{H}}$ and *D*_0_ should be computed in a range that is set as 0.5 to 6 and 10 to 25 by many experiments.

According to Section 2.1, the probability distribution of FFT of logarithm of coherent noise before inputting the adaptive Gaussian filter is approximately a Gaussian density. Thus, it can be written as

(14) $$P\left(x\right)=e^{-\frac{x^{2}}{2\sigma_{\varepsilon }^{2}}}$$

where $\sigma_{\varepsilon }^{2}$ is the variance of the noise and *ε*^2^ means the local quadratic residual between the original image $I_{0}$ and the smoothed image $I_{r_{H}}$ in space. 

The measure of information in bits can computed according to [14]. Thus, the description length of the residual is obtained as

(15) $$dl_{\varepsilon_{\sigma }}=\lambda (\frac{\varepsilon^{2}\sigma_{\varepsilon }^{2}}{2})$$

where λ=1/ln2, $dl_{I_{r_{H}}(u,v)}$, and $dl_{\varepsilon_{\sigma }(u,v)}$ have been defined, we use the sum of terms (12)–(15) to write the local description length as

(16) $$\begin{array}{ll} dl_{I_{0}(u,v)} & =dl_{I_{r_{H}}(u,v)}+dl_{I_{D_{0}}(u,v)}+dl_{\varepsilon_{\sigma }(u,v)}\\ & =n\mu \alpha (\beta^{2}r_{H}{}^{2}+D_{0})+\lambda (\frac{\varepsilon^{2}\sigma_{\varepsilon }^{2}}{2})\\ & =\eta (\beta^{2}rH^{2}+D_{0}^{2})+\varepsilon^{2} \end{array}$$

where $\eta =2\mu \alpha \beta^{2}/\lambda \sigma_{\varepsilon }^{2}$. In Equation (16), it is very easy to distinguish that *η* can be expressed the noise variance $\sigma_{\varepsilon }^{2}$, and it has effects on precision used to represent $I_{p}$.

Equation (16) is neither an edge stopping function or gradient threshold, and by controlling the rHmin, rHmax, $D_{0\min}$, and D0max the minimal and maximal amount of smoothness are obtained in the scale frequency. The filter shape with $r_{H}$ and *D*_0_ change as a function of MDL. 

### 3.3. Optimal Parameter Selection

As Equation (16) is defined, the local amount of smoothing can be computed directly using the $r_{H}$ and *D*_0_, and Gaussian filtering can be performed. The local description length at each (*u*,*v*) is computed through *I*_0_, $I_{p1},I_{p2},\ldots I_{pn}$. Then, we choose the minimal value of $dl_{I_{p}(u,v)}$ using the MDL principle at each location (*u*,*v*). The minimal value returns the optimal smoothness $r_{H}{}^{*}$ and $D_{0}{}^{*}$ in (*u*,*v*). The $dl_{I_{p}(u,v)}$ is minimum, and returns the maximum smoothing and the minimum residual with $r_{H}$ and *D*_0_. Finally, the output smoothed image in (*u*,*v*) is the intensity *I*_p_(*u*,*v*) at the selected $r_{H}{}^{*}(u,v)$ and $D_{0}{}^{*}(u,v)$. The parameters $r_{H}$ and *D*_0_ of the Gaussian filter at (*u*,*v*) allow us the possibility of making an adaptive filter. Each point (*u*,*v*) is blurred, as $r_{H}{}^{*}(u,v)$ and $D_{0}{}^{*}(u,v)$ can be expressed as

(17) $$I_{p(u,v)}=I_{0}(u,v)*\left(r_{H}{\left(u,v\right)}^{*}-r_{L}\right)\left[1-\exp\left\{-c{\left(\frac{D\left(u,v\right)}{D_{0}{}^{*}(u,v)}\right)}^{2}\right\}\right]+r_{L}$$

## 4. Results and Discussion

We conducted an experiment and simulation to verify the noise reduction performance. The sparse analysis model (SAM), the subspace-based spatial domain constraint method (SDC) and multi-channel logarithm with Gaussian denoising (MLG) were the results of some the latest studies on multiplicative noise filtering. Therefore, these three methods were compared with the proposed method.

The intensity of the sidelobe is almost equal to the intensity of the main lobe in the source localization system. This beam pattern can lead to the “confusion” phenomenon, which will cause large localization errors. The maximum sidelobe level (MSL) index has been widely used in source positioning systems [15] and antenna arrays to evaluate system performance and noise [16,17,18], such as sound localization systems. In electromagnetic imaging system, the presence of maximum sidelobes was the peak intensity value of coherent noise m^2^, and the main lobe intensity was the peak intensity value of the radiation source *w*^2^. It was defined as

(18) $$MSL=10\;\log_{10}(\frac{w^{2}}{m^{2}})$$

The sidelobe intensity was suppressed significantly, and the noise gain was simultaneously improved. A higher MSL value indicated a better noise suppression performance.

The dynamic range of the system describes the measurement between maximum and minimum values. To the electromagnetic imaging system, we can interpret dynamic range as the measurement between the lowest $I_{\min}$ and highest intensity $I_{\max}$ values of the electric field as follows:

(19) $$DR=10\;\log_{10}(\frac{I_{\max}}{I_{\min}})$$

### 4.1. Parameter Selection of the Adaptive Filter 

**Figure 5:**
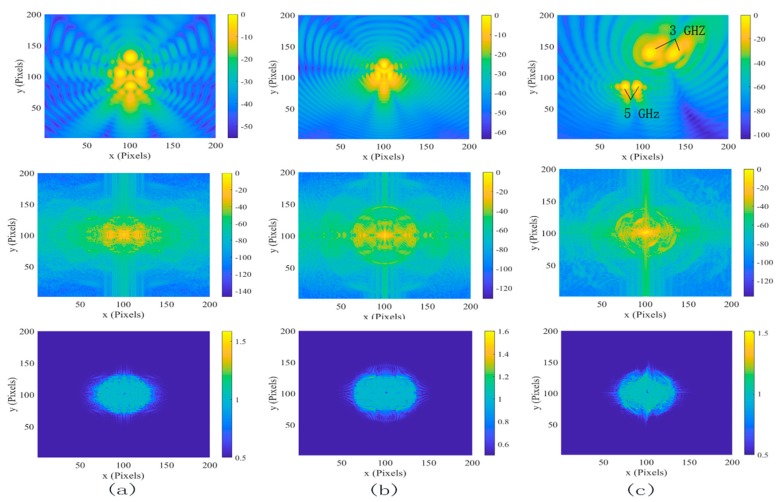
The original image of 4, 3, and 4 interference sources, and the logarithm spectral behavior of the coherent noise and Gaussian filter shape. (**a**) 2 GHz, (**b**) 4 GHz, and (**c**) 3 GHz and 5 GHz.

To verify the adaptive performance of the filter, the simulation was carried on several images at 2 GHz, 4 GHz, 3 GHz and 5 GHz, respectively. The MSL, DR, and maximum sidelobe before and after denoising were summarized in Table 1, in which the best results for denoising were expressed. Coherent noise was improved by 13 dB for a maximum value of MSL, and the dynamic range of the system increased by 76 dB after the proposed method was applied; this proved that a higher MSL value indicated a better noise suppression performance. The reason for this was that the MDL criterion was used to automatically adjust the value of $r_{H}$ and D_0_ to achieve the optimal filter according to the logarithm spectral behavior of the coherent noise of interference sources for different frequencies in Figure 5. The minimal and maximal amount of smoothness were controlled by the $r_{H}{}_{\min}$ and $r_{H}{}_{\max}$ from 0.5 to 6. The $D_{0}{}_{\min}$ and $D_{0}{}_{\max}$ were from 10 to 25. Notice that the Gaussian filter shape changed as a function of MDL by $r_{H}$ and *D*_0_. As a result, the local scale permitted effective noise elimination and accurate information preservation at the same time.

### 4.2. Test on Simulated Images

**Figure 6:**
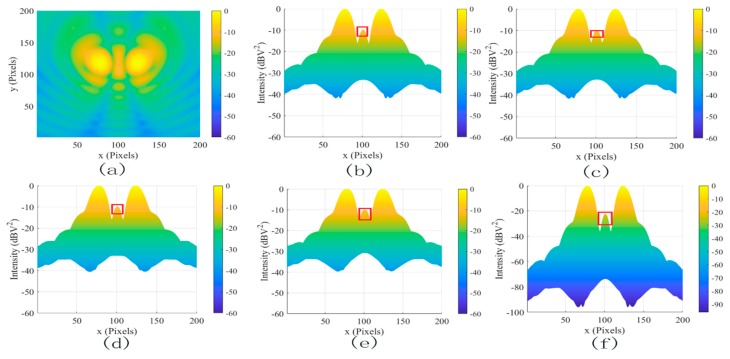
The filtered results for two dipole sources at 2 GHz. (**a**) The original image, (**b**) the lateral view of (**a**), (**c**) SDC, (**d**) MLG, (**e**) sparse analysis model (SAM), and (**f**) proposed.

**Figure 7:**
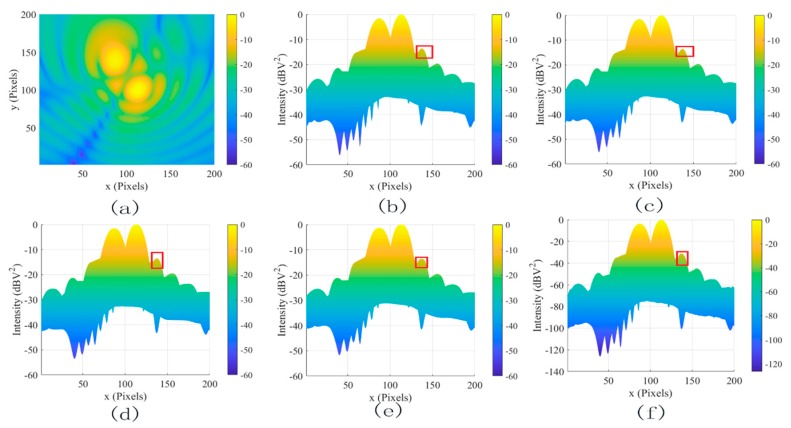
The filtered results for two dipole sources at 2 GHz. (**a**) The original image, (**b**) the lateral view of (**a**), (**c**) SDC, (**d**) MLG, (**e**) SAM, and (**f**) proposed.

Figure 6 illustrated the effects of different denoising methods for the two dipole sources at 2 GHz(a) at different positions. The SDC (c), MLG (d), and SAM methods (e) smooth the image, which is almost the same as (b). These methods did not work for coherent noise. In contrast, the proposed method (f) not only reduced the coherent noise from −10 dBv^2^ to −22 dBv^2^ but also increased the dynamic range of the system by 50 dB. To further test the coherent noise removal ability of the proposed method, two dipole sources at 2 GHz are shown in Figure 7a. In contrast, the coherent noise was reduced from −15 dBv^2^ to −30 dBv^2^ but also increased the dynamic range of the system by 65 dB. The results further proved the powerful denoising ability to coherent noise using the proposed method.

**Figure 8:**
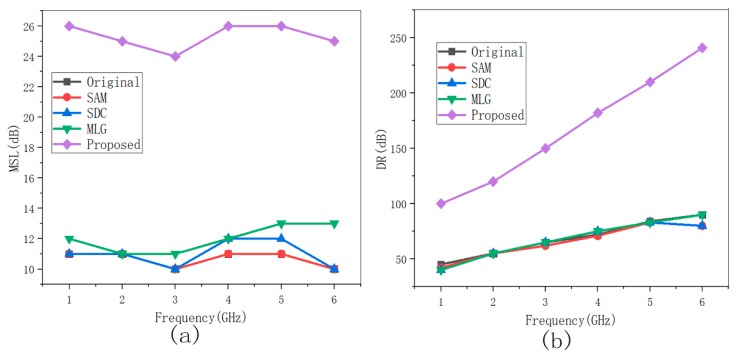
The MSL/DR results for two interference sources in Figure 6 at 1–6 GHz before and after denoising. (**a**) MSL with different frequencies and (**b**) DR with different frequencies.

**Figure 9:**
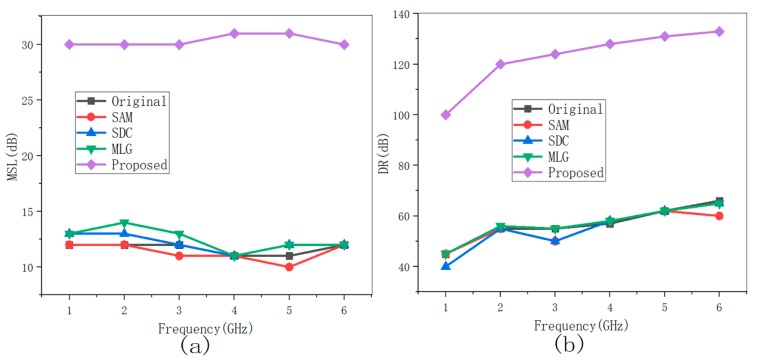
The MSL/DR results for two interference sources in Figure 7 at 1–6 GHz before and after denoising. (**a**) MSL with different frequencies and (**b**) DR with different frequencies.

The two dipole sources at different positions for 1 GHz to 6 GHz were adopted to evaluate the wideband performance of different denoising methods. The MSL and DR results of various methods are shown in Figure 8 and Figure 9. The SDC, SAM, and MLG methods almost have no promotion, because these methods had constraints to interference sources at different frequencies and positions. Significantly, the proposed method yielded more remarkable improvement on MSL by 12 dB and DR by 50 dB in Figure 6, and the MSL and DR improved by 15 dB and by 70 dB in Figure 7 compared with the others.

### 4.3. Test on Experimental Images

**Figure 10:**
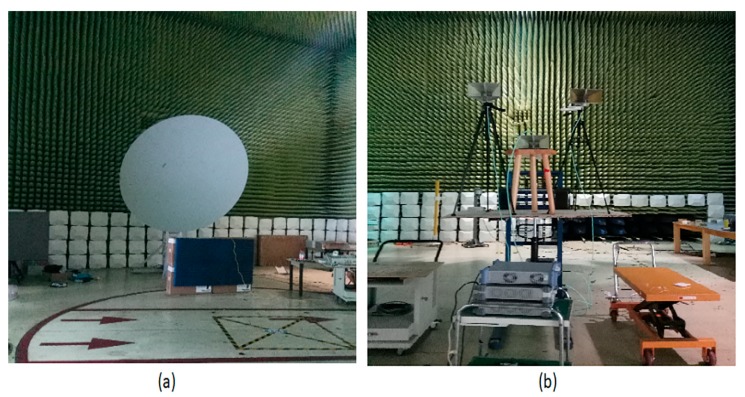
Settings of the experiment. (**a**) Reflective surface and (**b**) horn antennas.

**Figure 11:**
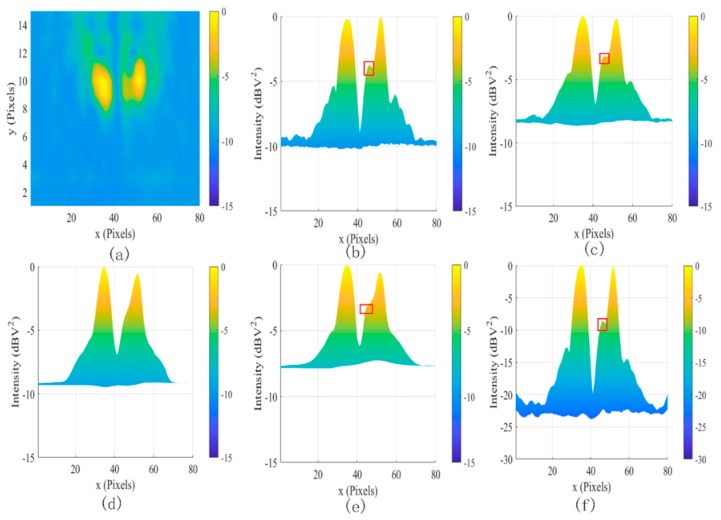
The filtered results for two horn antennas at 3 GHz. (**a**) The original image, (**b**) the lateral view of (a), (**c**) SDC, (**d**) MLG, (**e**) SAM, and (**f**) proposed.

**Figure 12:**
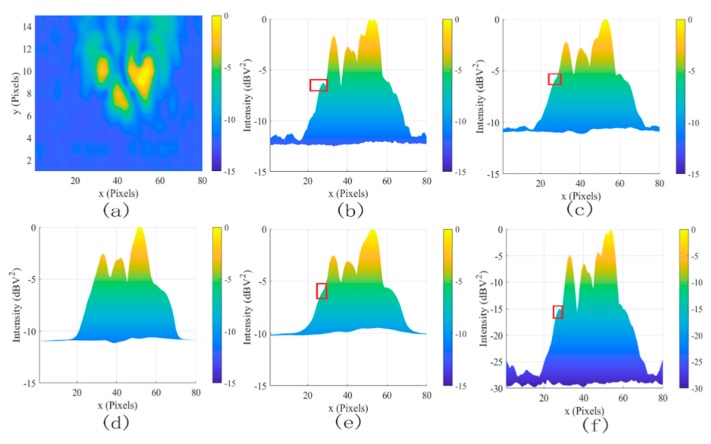
The filtered results for three horn antennas at 3 GHz. (**a**) The original image, (**b**) the lateral view of (a), (**c**) SDC, (**d**) MLG, (**e**) SAM, and (**f**) proposed.

To validate the effectiveness of the proposed method in practical measurement, the experiment in an anechoic chamber was built as shown in Figure 10a, using the two and three double-ridged horn antennas at 3 GHz (1 GHz–18 GHz) in a 5.2 m × 2.6 m rectangular area (b). The experiments were applied to a power divider to get a coherent signal. SDC (c) and SAM (e) methods could not achieve satisfying results in denoising task, and the value of DR was reduced. It can be seen that the peak intensity of coherent noise was removed in Figure 11d and in Figure 12d using the MLG method. However, the value of DR was reduced. In contrast, the proposed method fully considered the frequency domain property of coherent noise, which improved MSL and DR.

To completely illustrate time cost of the proposed method, two electromagnetic interference sources at 1 GHz, 3 GHz, and 6 GHz were tested and the bilinear interpolation was used to make the images different sizes. The average runtime of various denoising methods on different images is shown in Table 2. The proposed method had more promising time efficiency and higher performance than SAM, SDC, and MLG methods, which made it closer to the realization of real-time processing on terminal equipment.

## 5. Conclusions

In this work, an adaptive homomorphic filter is presented so that the natural logarithmic function is applied to add the multiplicative noise to the additive noise and directly compute the local Gaussian smoothing in terms of $r_{H}$ and *D*_0_. Our technique is not iterative, it is very stable and does not require any thresholds. The result of the experiments shows that the output of the adaptive filter can reduce the intensity of the coherent noise by 15 dB and could improve the dynamic range of the wideband electromagnetic imaging system by over 20 dB compared with the conventional narrowband denoising methods. 

This paper presents coherent noise suppression in a low-frequency wideband imaging system. It discusses the increase in the number of interference sources in electromagnetic images, as well as the fact that the numbers of interference sources in electromagnetic images are reduced due to the interference phenomenon. The electromagnetic imaging system can only measure the intensity of the electric field; it is very difficult to recover the phase of the interference source. In future work, we will optimize our method to solve the problem in which the numbers of interference sources are reduced without phase information. 

## Figures and Tables

**Table 1:** Maximum Sidelobe Level (MSL) and dynamic range (DR) results for electromagnetic interference sources (EMIS) at 2 GHz, 4 GHz, and 6 GHz before and after denoising.

Frequency (GHZ)	2	4	6
**Original** (dBv^2^)	−8	−8	−7
**Denoised** (dBv^2^)	−19	−21	−20
**MSL** (dB)	8/19	8/21	7/20
**DR** (dB)	48/105	51/120	64/140

**Table 2:** Average runtime (s) of various methods on different images at 1 GHz, 3 GHz, and 6 GHz.

Image Size	SAM	SDC	MLG	Proposed
90 × 90	9.164	3.5	5.218	2.197
150 × 150	25.231	7.834	6.243	3.721
180 × 180	37.412	10.93	7.265	5.292
220 × 220	54.042	15.46	7.846	7.244
